# Rapid Cytotoxicity of Antimicrobial Peptide Tempoprin-1CEa in Breast Cancer Cells through Membrane Destruction and Intracellular Calcium Mechanism

**DOI:** 10.1371/journal.pone.0060462

**Published:** 2013-04-05

**Authors:** Che Wang, Li-Li Tian, Song Li, Hui-Bing Li, Yang Zhou, He Wang, Qing-Zhu Yang, Li-Jie Ma, De-Jing Shang

**Affiliations:** 1 Liaoning Provincial Key Laboratory of Biotechnology and Drug Discovery, Liaoning Normal University, Dalian, China; 2 Department of Pharmacy, School of Chemistry and Chemical Engineering, Liaoning Normal University, Dalian, China; 3 Laboratory of Biophysics and Pharmacology, School of Physics and Optoelectronic Engineering, Dalian University of Technology, Dalian, China; 4 Faculty of Life Science, Liaoning Normal University, Dalian, China; Institute of Molecular and Cell Biology, Singapore

## Abstract

Temporin-1CEa is an antimicrobial peptide isolated from the skin secretions of the Chinese brown frog (Rana chensinensis). We have previously reported the rapid and broad-spectrum anticancer activity of temporin-1CEa in vitro. However, the detailed mechanisms for temporin-1CEa-induced cancer cell death are still weakly understood. In the present study, the mechanisms of temporin-1CEa-induced rapid cytotoxicity on two human breast cancer cell lines, MDA-MB-231 and MCF-7, were investigated. The MTT assay and the LDH leakage assay indicated that one-hour of incubation with temporin-1CEa led to cytotoxicity in a dose-dependent manner. The morphological observation using electronic microscopes suggested that one-hour exposure of temporin-1CEa resulted in profound morphological changes in both MDA-MB-231 and MCF-7 cells. The membrane-disrupting property of temporin-1CEa was further characterized by induction of cell-surface exposure of phosphatidylserine, elevation of plasma membrane permeability and rapid depolarization of transmembrane potential. Moreover, temporin-1CEa evoked intracellular calcium ion and reactive oxygen species (ROS) elevations as well as collapse of mitochondrial membrane potential (Δφm). In summary, the present study indicates that temporin-1CEa triggers rapid cell death in breast cancer cells. This rapid cytotoxic activity might be mediated by both membrane destruction and intracellular calcium mechanism.

## Introduction

Numerous chemotherapeutic agents have been developed for cancer treatment, including antimetabolites, DNA alkylating drugs, and hormone agonists/antagonists. A major limitation inherent to most of these conventional anticancer drugs is their inability to distinguish between cancer cells and proliferating normal cells and therefore, leading to severe side-effects and dose limitations. Moreover, cancer cells can develop resistance to these drugs that is mediated by the overexpression of multidrug-resistance proteins that pump the drugs out of cells and thus render the drugs ineffective [1]. Recently, antimicrobial peptides (AMPs, also termed host defense peptides) have been shown to exert potent antitumor effects both in vitro and in vivo and received attention as new class anticancer molecules [2]–[5]. These peptides have several advantages over currently used anticancer therapeutics, such as selective cytotoxicity for cancer cells, bypass of the multidrug-resistance mechanism, and synergism effects in combination therapy [6].

Many AMPs damage the cellular membrane as part of their killing mechanism. Although the interactions that take place between AMPs and the outer membrane leaflet of neoplastic eukaryotic cells are not completely understood, the mechanism by which AMPs interacts with microbial cytoplasmic membranes may provide important clues to this process. The net negative charge that is conferred upon many cancer cells as a result of differential branching and sialic acid content of N-linked glycans associated with transmembrane glycoproteins [7], as well as the elevated cell surface anionic molecules such as phosphatidylserine [8], [9] and O-glycosylated mucins [10], [11], is believed to promote electrostatic interactions with AMPs at the cancer cell surface. Then the membrane-bound AMPs disrupted cell membrane through pore formation or membrane destabilization [12]. Besides the direct membrane-destructing effect, some researchers have suggested that AMPs might exert cytolytic activity against cancer cells through ion-permeable channel formation in the cell membrane [13] or other non-membranolytic intracellular actions [14]–[16].

Temporin-1CEa is a cationic amphiphilic antimicrobial peptide isolated from the skin secretions of the Chinese brown frog (*Rana chensinensis*). We have recently reported that temporin-1CEa exhibits rapid cytotoxic activity against both microorganisms and human cancer cells [17], [18]. In the present study, we have therefore continued our investigations for elucidation of anticancer mechanisms of temporin-1CEa. The study presents the anticancer activity of temporin-1CEa against two human breast cancer cell lines MDA-MB-231 and MCF-7 utilizing MTT assay and LDH leakage assay for cell death, together with scanning electron microscopy (SEM) and transmission electron microscopy (TEM) for providing insights into morphological changes. In addition, cell-membrane permeability is determined using flow cytometry and an annexin-V-FITC/propidium-iodide protocol. The intracellular calcium ion and reactive oxygen species (ROS) concentrations and mitochondrial membrane potential were also evaluated. The results presented here may provide new insights helping to understand the direct membrane-destruction effect and intracellular mechanisms of temporin-1CEa in breast cancer cells.

## Results

### Temporin-1CEa Induces Breast Cancer Cell Death

**Figure 1:**
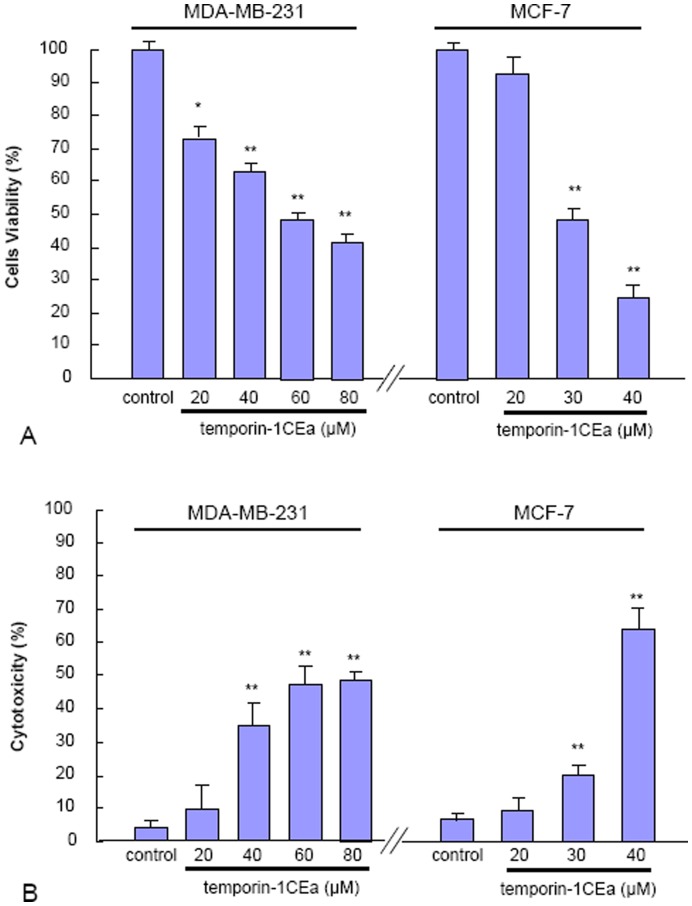
Temporin-1CEa-induced MDA-MB-231 and MCF-7 cells death. The cells were treated with various concentrations of temporin-1CEa for one hour. Cell viability or cytotoxicity was determined using the MTT method (A) or LDH leakage assay (B). Each bar represents the mean value from three determinations with the standard deviation (SD). Data (mean ± SD) with asterisk significantly differ (*p<0.05; **p<0.01) between treatments.

As shown in Fig. 1, both MTT assay and LDH leakage assay indicated that treatment of cancer cells with temporin-1CEa induced cell death in a concentration-dependent manner. The formazan production (optical density) measured in MTT assay (Fig.1A) was reduced after one hour of incubation with peptides; meanwhile, the extracellular LDH activity value (optical density) was enhanced in LDH leakage assay (Fig.1B). Moreover, the in vitro cytotoxicity assay also indicated that MCF-7 cancer cells were more vulnerable to the temporin-1CEa-induced cytotoxicity than MDA-MB-231 cells. For instance, after one hour exposure to 40 µM temporin-1CEa, the percentages of cells viability and cytotoxicity as assessed by MTT assay and LDH leakage assay were 22% ±4% and 61% ±7% for MCF-7 cell line, and were 61% ±2% and 32% ±8% for MDA-MB-231 cell line.

### Temporin-1CEa Induces Morphological Changes

**Figure 2:**
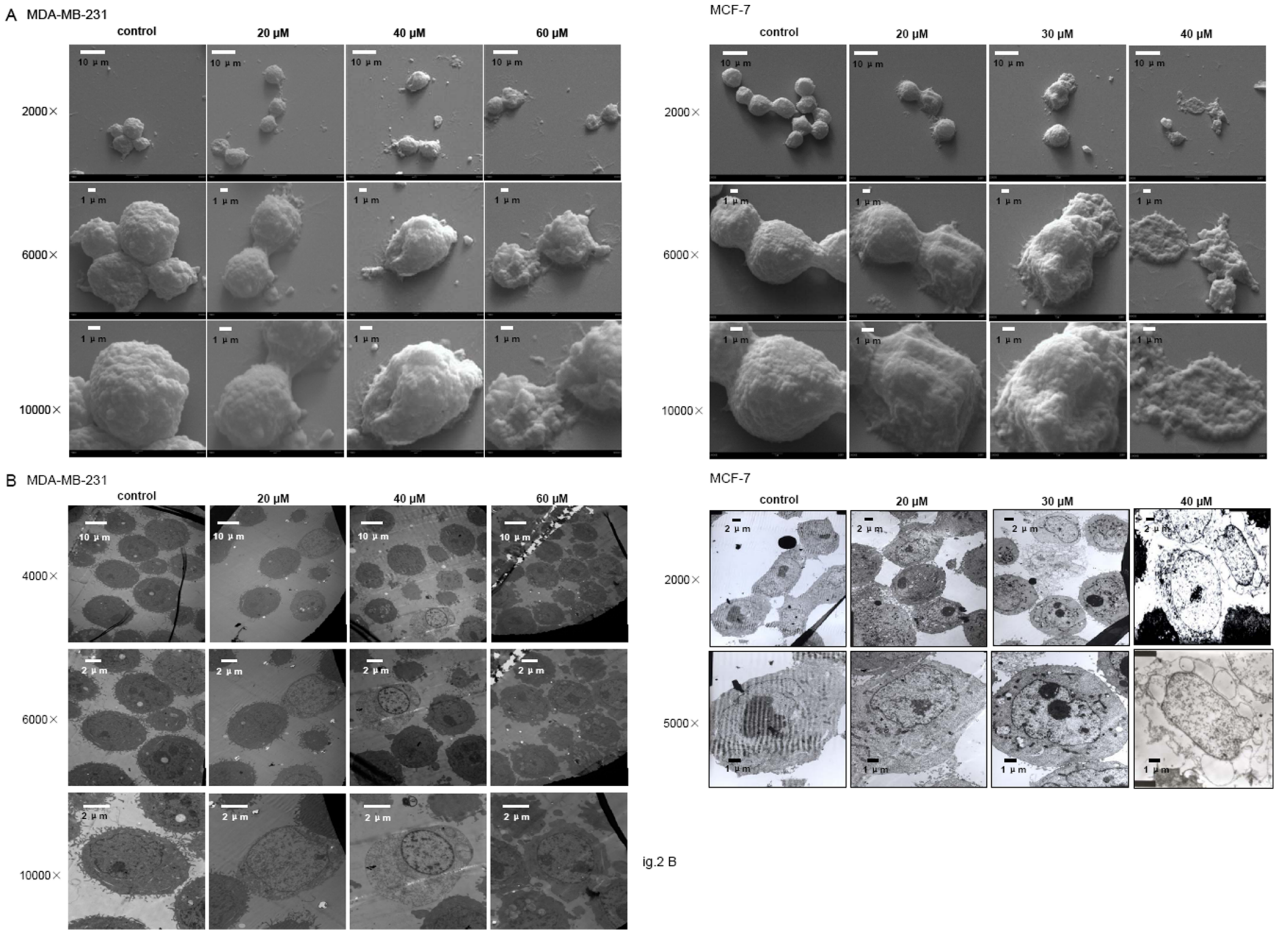
Morphological changes of MDA-MB-231 and MCF-7 cells upon one-hour exposure to temporin-1CEa. SEM (A) and TEM (B) evaluation of breast cancer cells treated with temporin-1CEa.

Morphological examination via scanning electron microscopy (Fig. 2A) or transmission electron microscopy (Fig. 2B) revealed that one hour incubation of various concentrations of temporin-1CEa induced dramatic morphological changes in both MDA-MB-231 and MCF-7 breast cancer cells. While untreated control cells showed an intact membrane and smooth surface, the temporin-1CEa-treated cancer cells membrane were shriveled, invaginated and disrupted, which may in turn resulted in irreversible cytolysis and finally death of the target cells.

### Temporin-1CEa Induces Cell Surface Exposure of Phosphatidylserine and Disruption of Plasma Membrane Integrity in MDA-MB-231 Cells

**Figure 3:**
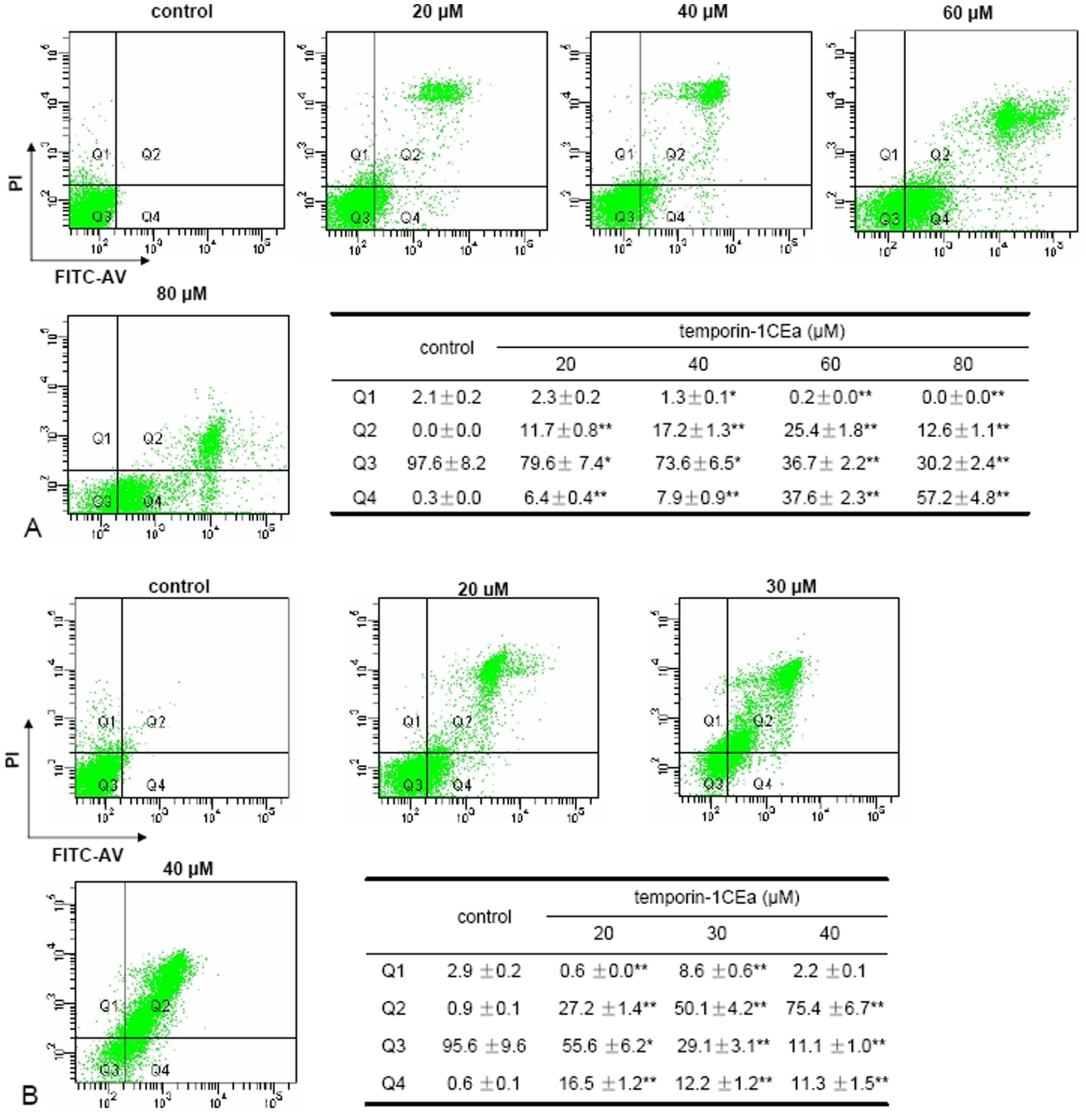
Temporin-1CEa induced loss of membrane integrity and phosphatidylserine exposure in two human breast cancer cell lines. MDA-MB-231 cells (A) or MCF-7 cells (B) were incubated with various concentrations of temporin-1CEa for one hour and then were stained with Annexin-V-FITC/PI. Fluorescence intensity was determined using flow cytometry. Each bar represents the mean value from three determinations with the standard deviation (SD). Data (mean ± SD) with asterisk significantly differ (*p<0.05; **p<0.01) between treatments.

Phosphatidylserine (PS) exposure on the surface of cells has been considered a characteristic feature of membrane disruption or cell death [19]. When the plasma membrane loses its integrity, PS originally exist in the inner leaflet of the plasma membrane will be exposed to outer surface. PS can be detected and observed via FITC-coupled Annexin-V which specifically binds to PS molecule when exposed at the cell surface. PI, a membrane impermeant dye, only enters membrane compromised cells, after which the fluorescence of this probe is enhanced by 20–30-fold because of its binding to nucleic acids. In the present study, MDA-MB-231 or MCF-7 cancer cells were incubated with temporin-1CEa for one hour. After treatment with peptides, the cells were stained with FITC-Annexin V and PI. The cell-surface phosphatidylserine (PS) exposure and plasma membrane integrity was analyzed using FACSCanto flow cytometer (BD Biosciences). Cells were sorted as: viable cells (FITC-annexin V negative and PI negative, Q3 quadrant), cells with membrane lipid asymmetry and PS exposure (FITC-annexin V positive, Q4 quadrant), and cells with interrupted membrane integrity (FITC-annexin V positive and PI positive, Q2 quadrant). The FACS analysis indicated that while the viable cells captured in Q3 quadrant was reduced, the cells captured in Q2 or Q4 quadrant was increased (Fig. 3A for MDA-MB-231 and Fig.3B for MCF-7 cells, respectively). These results indicated that temporin-1CEa could affect cancer cells viability by disrupting their membrane integrity (as shown by the cell surface PS exposure) and increasing membrane permeability (as indicated by uptake of PI into cells). In addition, the cells sorting data also suggested two cancer cell lines exerted different response manners to temporin-1CEa exposure. Consistent with the in vitro cytotoxicity assay, MCF-7 cells were more susceptible than MDA-MB-231 cells as indicated by the lower levels of viable cells in Q3 quadrant. Moreover, the temporin-1CEa-treated MCF-7 cells seemed to be more permeable for PI as indicated by higher Q2 values (Fig. 3B), while MDA-MB-231 cells preferred to be affected on the cell surface as indicated by higher Q4 value (Fig. 3A).

### Temporin-1CEa Induces Enhancement of Membrane Permeability

**Figure 4:**
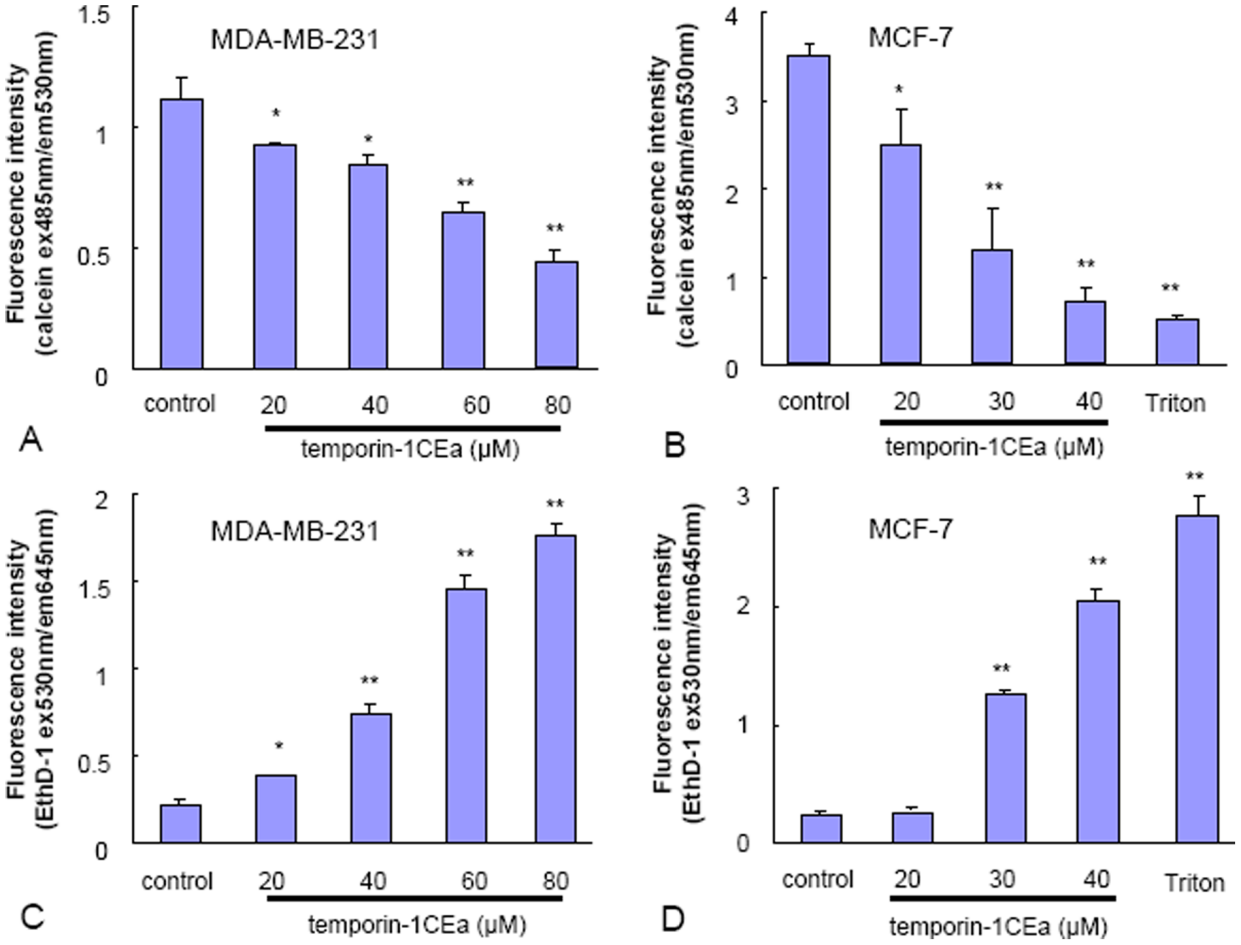
Temporin-1CEa induces enhancement of membrane permeability in MCF-7 and MDA-MB-231 cells using calcein AM/EthD-1 staining. Breast cancer cells were exposed to various concentrations of temporin-1CEa or were left untreated for 60 min, then the medium was removed, and 20 µl of dye containing 2 µM calcein AM and 4 µM EthD-1 was added for 30 min in the dark. The fluorescence intensity was distinguished by FACS analysis. Each bar represents the mean value from three determinations with the standard deviation (SD). Data (mean ± SD) with asterisk significantly differ (*p<0.05; **p<0.01) between treatments.

To confirm the membrane permeablizing effect of temporin-1CEa on MDA-MB-231 and MCF-7 cells, calcein AM and ethidium homodimer (EthD-1) were used. Live cells have intracellular esterases that convert nonfluorescent, cell-permeable calcein AM to the intensely fluorescent green calcein, which is retained within the live cells with intact membrane. EthD-1 is excluded by the intact plasma membrane of live cells. However, in membrane-disrupted or dead cells with enhanced membrane-permeability, EthD-1 enters into intracellular space of cells and produces bright-red fluorescence when bound to nucleic acids. Therefore, an increased fluorescence intensity of EthD-1 or a decreased fluorescence intensity of calcein means enhanced membrane permeability and interrupted membrane integrity. As shown in Fig. 4, after one-hour exposure of temporin-1CEa, the fluorescence intensity of calcein was reduced (Fig. 4A-B); meanwhile, the fluorescence intensity of EthD-1 was increased in cells (Fig. 4C-D). These results suggested that temporin-1CEa disrupted the cell membranes of MDA-MB-231 and MCF-7 cells leading to an increase in membrane permeability. Moreover, in the absence or presence of temporin-1CEa, the MDA-MB-231 cells showed a lower permeability for the membrane-permeable calcein AM than MCF-7 cells, as indicated by the lower level of intracellular calcein fluorescence intensity.

### Distributions of Temporin-1CEa in MDA-MB-231 Cells

**Figure 5:**
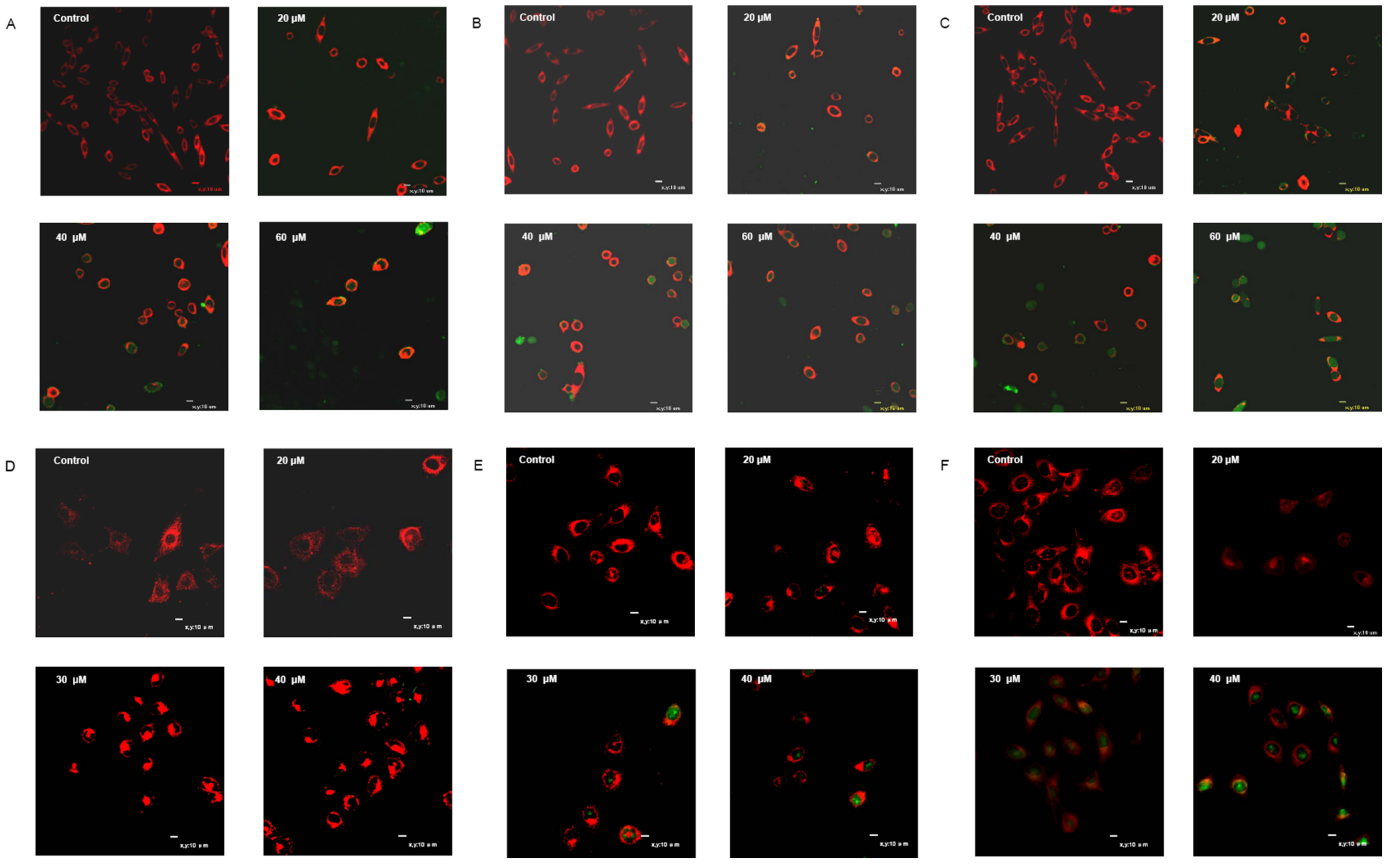
Laser scanning confocal microscopy images of MDA-MB-231 and MCF-7 cells treated with FITC-labeled temporin-1CEa for 5 min (A: MDA-MB-231; D: MCF-7), 10 min (B: MDA-MB-231; E: MCF-7) and 60 min (C: MDA-MB-231; F: MCF-7).

Since temporin-1CEa disrupted cell membranes, we further determined whether temporin-1CEa itself has the potential to transfuse or influx into MDA-MB-231 and MCF-7 cells to trigger intracellular events. The peptides were labeled with FITC and co-cultured with MDA-MB-231 or MCF-7 cells for one hour. Myelin and other lipophilic areas on cell membrane were stained with the red-orange fluorescent tracker DiI. The dynamic changes of distributions of the FITC-labeled-temporin-1CEa were traced using laser scanning confocal microscopy. The results indicated that the extracellular peptides transferred through the cell membrane into the intracellular space, characterized by an increase of intracellular green fluorescence (Fig. 5). In detail, at lower concentration (20 µM), the fluorescence imaging indicated that most of the peptides were prevented from cell membranes as shown by the less intracellular green fluorescence. However, at higher concentrations (40–60 µM in MDA-MB-231 and 30–40 µM in MCF-7 cells), temporin-1CEa disrupted the membrane integrity and caused rapid peptides influx into cells (as shown by increased intracellular green fluorescence from FITC, even after a 5 or 10 min short-term peptide treatment, Fig.5).

### Transmembrane Potential Depolarization Induced by Temporin-1CEa

**Figure 6:**
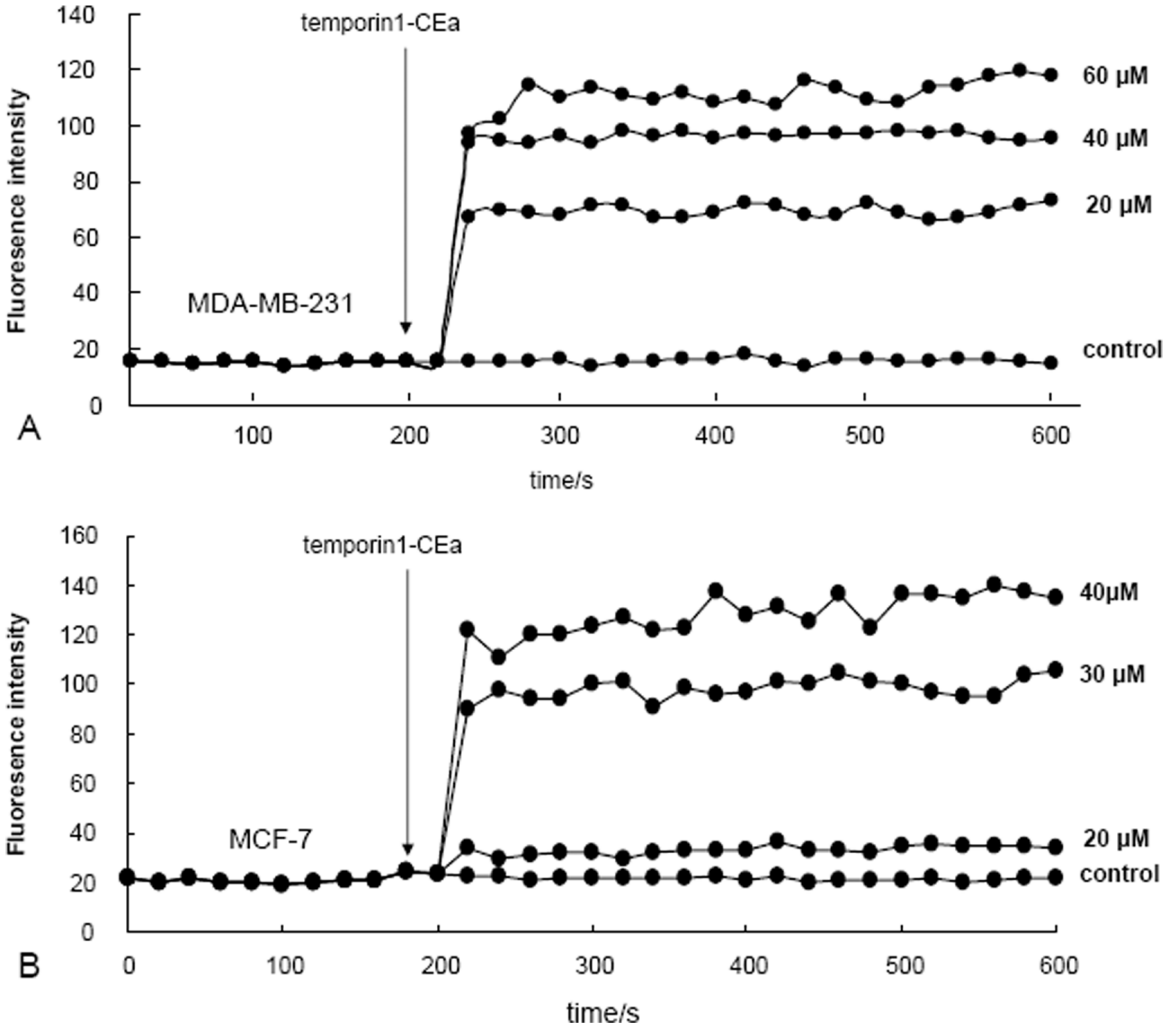
Transmembrane potential of breast cancer cells after peptides treatment. The transmembrane potential depolarization of cancer cells (A: MDA-MB-231 and B: MCF-7) were determined using the anionic dye, DiBAC_4_(3).

MDA-MB-231 and MCF-7 cells were incubated with DiBAC_4_(3), one anionic and membrane-potential-sensitive dye. Depolarization of cell membranes leads to an uptake of DiBAC_4_(3) inside the cells, resulting in an increased fluorescent signal. As seen in Fig. 6, one hour exposure of temporin-1CEa to MDA-MB-231 (Fig. 6A) or MCF-7 (Fig. 6B) cells led to an immediate and dramatic increase in the fluorescence intensity of DiBAC_4_(3), which was not observed in control cells. This rapid and dose-dependent depolarization of transmembrane potential might be due to the enhancement of membrane permeability and formation of ion fluxes through the disrupted cancer cell membranes.

### Temporin-1CEa Increases Cytosolic Calcium Level

**Figure 7:**
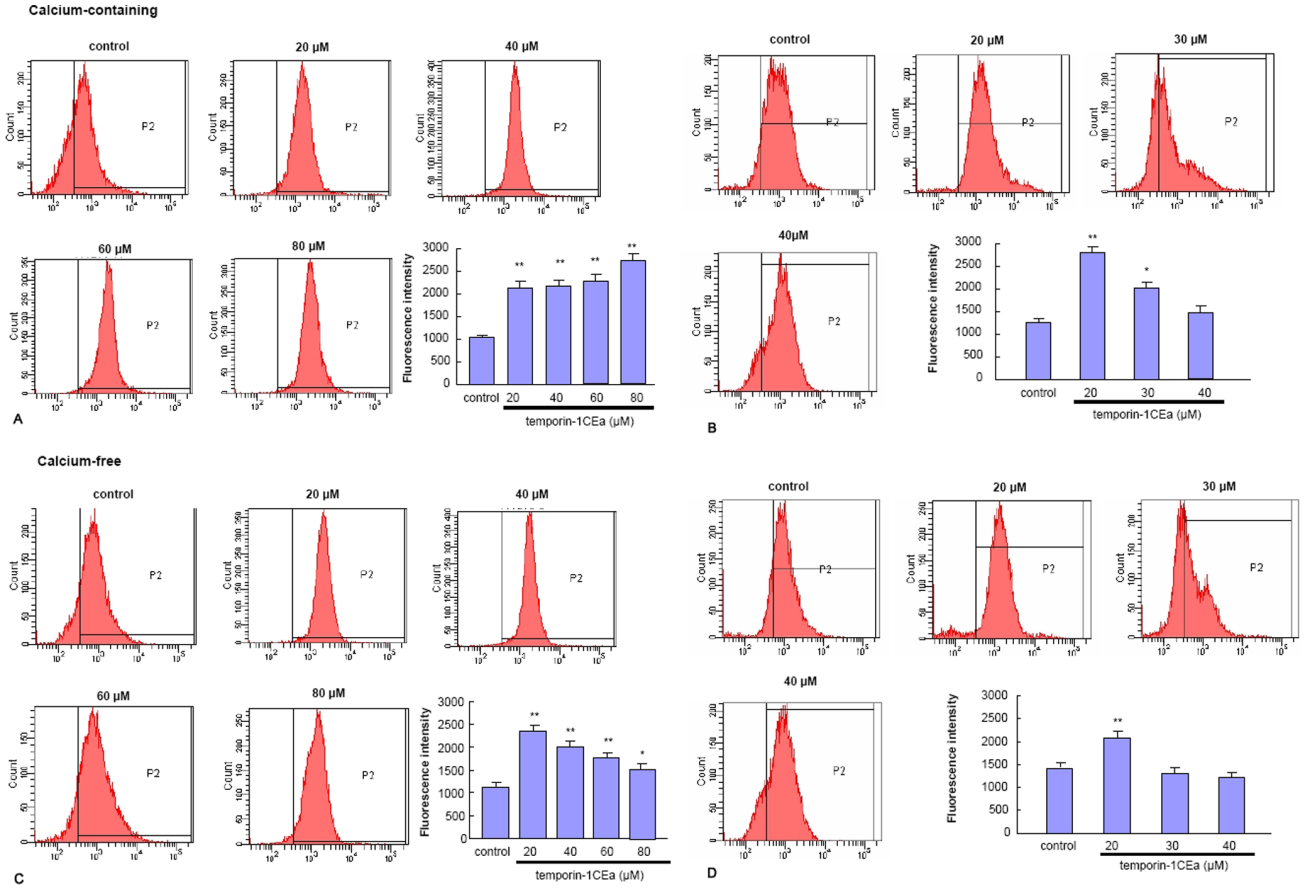
Elevation of intracellular calcium concentration in MDA-MB-231 or MCF-7 cells following temporin-1CEa treatment in either a calcium-containing solution (A-B) or calcium-free solution (C–D). Fluo3-AM green fluorescence was used for evaluation of the cytosolic calcium levels. Each bar represents the mean value from three determinations with the standard deviation (SD). Data (mean ± SD) with asterisk significantly differ (*p<0.05; **p<0.01) between treatments.

To clarify the possible involvement of calcium in the peptide-induced membrane potential depolarization, the intracellular Ca^2+^ concentration was evaluated in either a calcium-containing or a calcium-free situation. In the calcium-containing situation, FACS analysis indicated that incubation of temporin-1CEa on MDA-MB-231 (Figs. 7A) or MCF-7 cells (Fig. 7B) led to an increase of intracellular Ca^2+^ concentration. This upregulation of the Ca^2+^ content might be due to an influx of extracellular Ca^2+^, and/or an endogenous Ca^2+^ release from the intracellular calcium stores.

To further clarify whether temporin-1CEa-caused intracellular Ca^2+^ elevation was induced by an endogenous Ca^2+^ release or an extracellular Ca^2+^ influx, the intracellular Ca^2+^ concentration was determined in a calcium-free situation. FACS analysis demonstrated that one-hour treatment of cancer cells with temporin-1CEa under calcium-free medium also caused significant upregulations of cytosolic Ca^2+^ concentration. This upregulation of Ca^2+^ level was due to the calcium leakage from intracellular stores because of the calcium-free medium (Figs. 7C-7D). However, the up-regulation of intracellular Ca^2+^ concentration was declined in cells treated with a higher dose of temporin-1CEa (at 40–80 µM for MDA-MB-231 and 30–40 µM for MCF-7 cells), which might be due to Ca^2+^ efflux induced by transmembrane Ca^2+^ gradient or due to the seriously disrupted membrane structure during the late phage of peptides exposure. These results suggested that temporin-1CEa could induce an intracellular Ca^2+^ overload and that this effect was independent of extracellular Ca^2+^ concentrations.

### Temporin-1CEa Disrupts the Mitochondrial Membrane Potential (Δφm)

**Figure 8:**
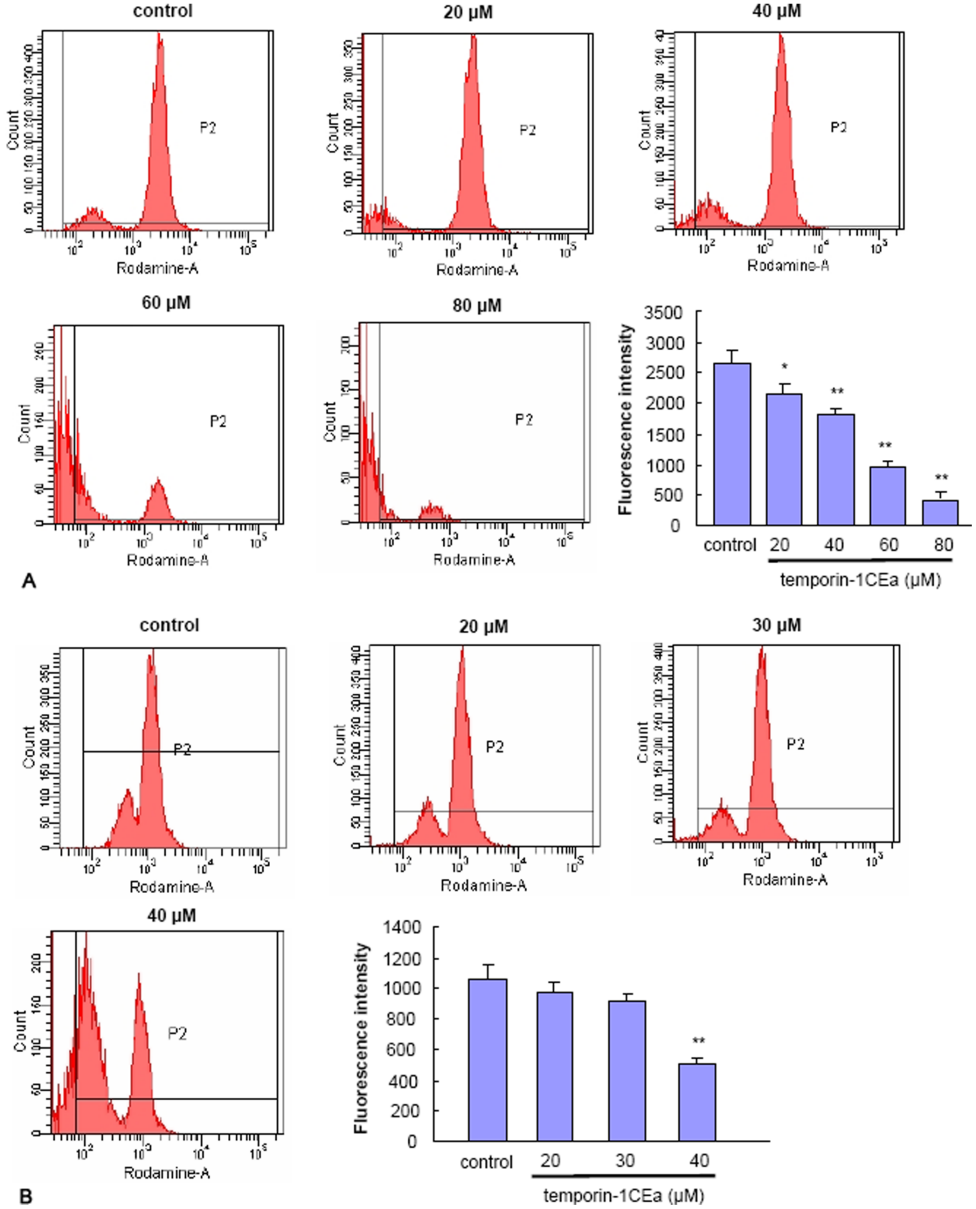
Disruption of mitochondrial membrane potential in MDA-MB-231 (A) and MCF-7 cells (B) after temporin-1CEa exposure. Mitochondrial membrane potential was measured using the cell-permeable fluorescent cationic dye rhodamine 123 by flow cytometry. Each bar represents the mean value from three determinations with the standard deviation (SD). Data (mean ± SD) with asterisk significantly differ (*p<0.05; **p<0.01) between treatments.

Temporin-1CEa disrupted the membrane integrity and uptake into cells. Given the negative charge of mitochondrial membranes, mitochondria are possibly the preferential intracellular structural target for internalized temporin-1CEa. Moreover, the elevated intracellular Ca^2+^ concentration is usually preceded or accompanied with a reduction in the Δφm. To address whether temporin-1CEa-induced calcium overload is associated with the changes of Δφm, MDA-MB-231 or MCF-7 cells were treated with temporin-1CEa and were stained with rhodamine 123 to assess the Δφm. Treatment with temporin-1CEa produced a remarkable loss of Δφm at higher concentrations (at 60–80 µM for MDA-MB-231, Fig. 8A; and 40 µM for MCF-7 cells, Fig. 8B).

### ROS Generation in Temporin-1CEa-treated Cancer Cells

**Figure 9:**
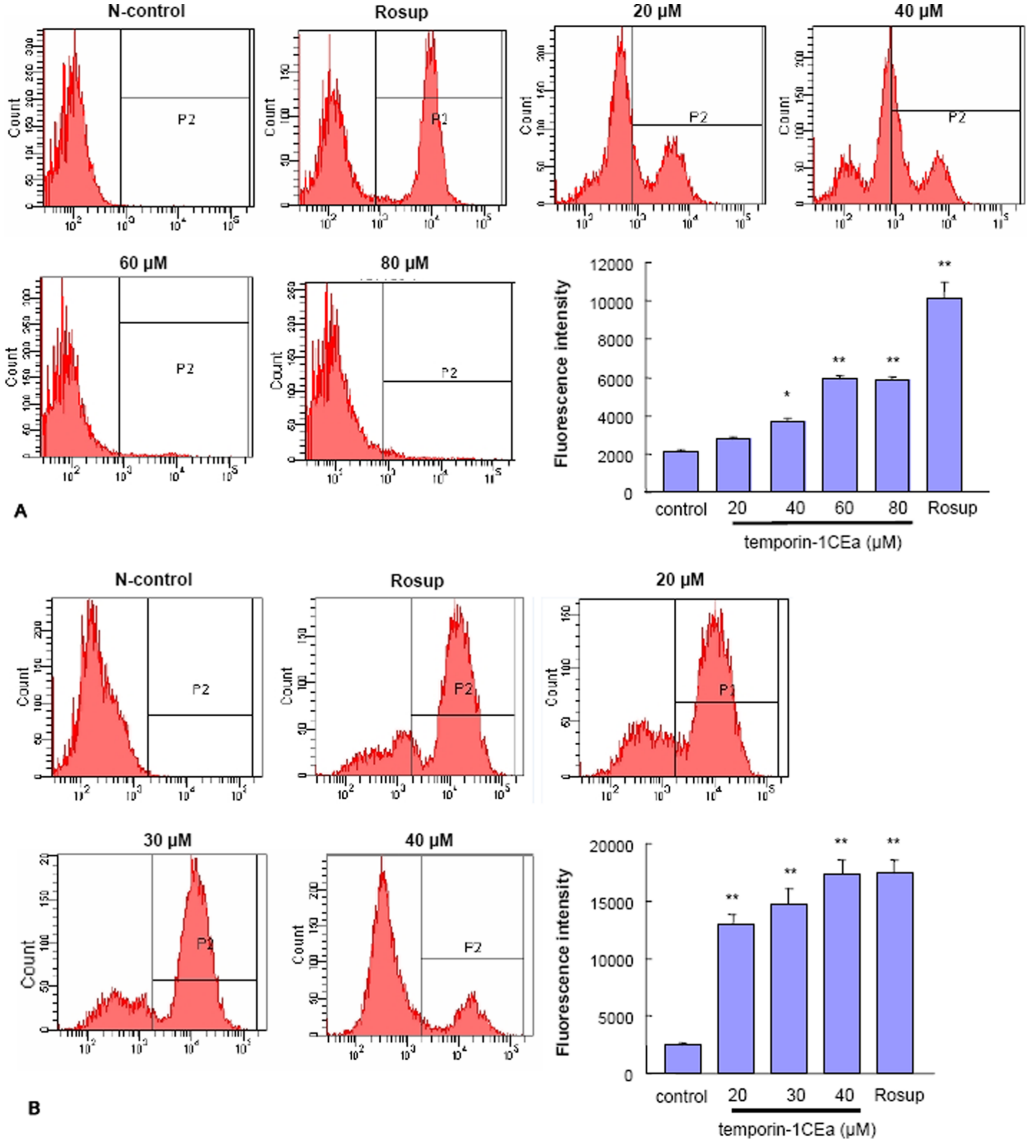
Release of intracellular ROS in MDA-MB-231 (A) and MCF-7 (B) cells after temporin-1CEa treatment. ROS production was measured by FACS analysis using a sensitive free-radical indicator, 2′,7′-dichlorofluorescin-diacetate (DCFH-DA). Each bar represents the mean value from three determinations with the standard deviation (SD). Data (mean ± SD) with asterisk significantly differ (*p<0.05; **p<0.01) between treatments.

Temporin-1CEa-induced intracellular ROS generation was evaluated using intracellular peroxide-dependent oxidation of DCFH-DA to form fluorescent DCF. DCF fluorescence was detected after cells were treated with temporin-1CEa for 60 min. The group with absence of temporin-1CEa was a negative control, while Rosup-induced intracellular peroxide production was used as a positive control. The results indicated that ROS production was significantly increased upon treatment with temporin-1CEa compared with negative control (Fig. 9). Moreover, while two cell lines with absence of temporin-1CEa exposure showed a similar ROS basal level, temporin-1CEa exposure generated a higher level of ROS production in MCF-7 cells (Fig. 9B) than in MDA-MB-231 cells (Fig. 9A).

## Materials and Methods

### Cell Culture and Peptide Treatment

Two human breast cancer cell lines, MDA-MB-231 and MCF-7, were obtained from the Cell Bank of Chinese Academy of Sciences (Shanghai, China). The breast cancer cells were cultured in medium (MDA-MB-231 cells were in L15 medium, for MCF-7 cells were RPMI-1640) containing 10% fetal bovine serum, 2 mM l-glutamine, 100 U/ml penicillin and 100 µg/ml streptomycin in a humidified incubator at 37°C with 5% CO_2_ [18], [20], [21]. We have previously reported that one hour of temperin-1CEa incubation exerts cytotoxicity to MDA-MB-231 and MCF-7 cells with IC50 values of 31.78 and 63.26 µM respectively [18]. Therefore, in the present study, the concentrations of temporin-1CEa were ranged from 20 to 40 µM for MCF-7 and from 20 to 80 µM for MDA-MB-231 cell line.

### *In vitro* Cytotoxicity Assay

MDA-MB-231 or MCF-7 breast cancer cells were cultured in 96-well plates at 5×10^4^ cells/ml (0.1 ml/well) then incubated with various concentrations of temporin-1CEa for one hour. The cells viability or cytotoxicity was measured using the 3-(4,5-dimethylthiazol-2-yl)-2,5-diphenyl tetrazolium bromide (MTT) assay or lactate dehydrogenase (LDH) leakage assay.

### Morphological Analysis using Electronic Microscopes

Some AMPs can target nonpolar lipid cell membranes resulting in finally death of the target cells. Therefore, in the present study, the morphological changes of MDA-MB-231 and MCF-7 cells after one-hour of temporin-1CEa treatment were imaged by a scanning electronic microscope and a transmission electron microscope using standard protocols [22].

### Assessment of Cell-surface Phosphatidylserine Exposure and Plasma Membrane Integrity using FITC-annexin-V/propidium Iodide (PI) Staining

MDA-MB-231 or MCF-7 cancer cells were seeded in a 96-well plate and incubated with various concentrations of temporin-1CEa or were left untreated (control) for 60 min. After treatment with peptides, the cells were stained with FITC-annexin V and PI according to manufacturer’s instructions (FITC-Annexin V Apoptosis Detection Kit, BD Biosciences). The cell-surface phosphatidylserine (PS) exposure and plasma membrane integrity was analyzed using FACSCanto flow cytometer (BD Biosciences). Cells were sorted as: viable cells (FITC-annexin V negative and PI negative, Q3 quadrant), cells with membrane lipid asymmetry and PS exposure (FITC-annexin V positive, Q4 quadrant), and cells with interrupted membrane integrity (FITC-annexin V positive and PI positive, Q2 quadrant).

### Cell Membrane Permeability Assay using Calcein AM and Ethidium Homodimer (EthD-1) Staining

The cell membrane integrity and permeability was determined using a two-color fluorescence assay with two probes, EthD-1 and calcein AM. MDA-MB-231 or MCF-7 cells were seeded into 96-well plates at 5×10^4^ cells/ml. After exposure to various concentrations of temporin-1CEa for 60 min, the medium was removed, and 20 µl of dye containing 2 µM calcein AM and 4 µM EthD-1 was then added for 30 min in the dark. The fluorescence intensity was distinguished by FACS analysis with Ex485 nm/Em530 nm for calcein and Ex530 nm/Em645 nm for EthD-1.

### FITC-labeled Peptides Uptake

MDA-MB-231 or MCF-7 cancer cells were incubated with various concentrations of FITC-labeled temporin-1CEa for 5, 10 or 60 min. Myelin and other lipophilic areas on cell membrane were stained with the red-orange fluorescent tracker DiI. The FITC-labeled peptides were traced and recorded at each time point using laser scanning confocal microscopy.

### Transmembrane Potential Measurements

Cell transmembrane potential depolarization were measured using the membrane potential sensitive dye, bis-(1,3-dibutylbarbituric acid) trimethin eoxonol [DiBAC_4_(3)]. Upon depolarization of the transmembrane potential, the DiBAC_4_(3) enters the cell and binds to protein molecules to give enhanced fluorescence. In contrast, fluorescence intensity was reduced when the transmembrane potential was hyperpolarized. As previously described [23], after incubation with 2 µM DiBAC_4_(3) for 10 min at 37°C, the cells were subjected to time scanning using a fluorescence spectrophotometer (Varioskan Flash, Thermo Scientific) with Ex488 nm/Em518 nm. When the fluorescence intensity was stable, the cells were treated with temporin-1CEa or sterile-deionized water. Membrane depolarization was monitored by observing the changes in the intensity of fluorescence emission of DiBAC_4_(3).

### Cytosolic Calcium Ion (Ca^2+^) Concentration Determination

To detect intracellular Ca^2+^ levels, the Ca^2+^-specific fluorescent dye, Fluo3-AM, was loaded into MDA-MB-231 or MCF-7 cells using a modified procedure adapted from the manufacturer (Beyotime, China). Briefly, cells were incubated for 30 min at 37°C with 4 µM Fluo3-AM in Hank’s buffered salt solution (HBSS) with or without 1.3 mM Ca^2+^. The cells were then washed two times with fresh HBSS and incubated in HBSS at room temperature. The Fluo3-AM green fluorescence (Ex488 nm/Em526 nm) was proportional to intracellular Ca^2+^ concentrations. The changes of Fluo3-AM intensity in response to the peptides exposure were monitored using flow cytometry.

### Measurement of Mitochondrial Membrane Potential

The changes of mitochondrial membrane potential (Δφm) were measured using rhodamine 123 fluorescence. Rhodamine 123 is a cationic lipophilic fluorochrome whose distribution to the mitochondria matrix correlates with the Δφm [24]. One hour after exposure to temporin-1CEa, cells were loaded with 10 µM rhodamine 123 and incubated at 37°C for 30 minutes in the dark. Cells were then harvested, washed, and resuspended in PBS and analyzed immediately using flow cytometry with the Ex488 nm/Em525 nm.

### Determination of Reactive Oxygen Species (ROS) Production

The intracellular accumulation of ROS was determined using a sensitive free-radical indicator, 2′,7′-dichlorofluorescin-diacetate (DCFH-DA). The nonfluorescent DCFH can be oxidized by ROS to form green fluorescent molecule, 2′,7′-dichlorofluorescein (DCF). Temporin-1CEa-treated MCF-7 or MDA-MB-231 cells were incubated with 25 µM DCFH-DA in darkness for 30 min. After incubation, cells were collected, washed with PBS, resuspended in PBS and then subjected to flow cytometry for analysis.

## Discussion

In the present and our previous work, we have shown that temporin-1CEa, an AMP from the skin secretions of the Chinese brown frog (*Rana chensinensis*), is a potent antitumor agent. AMPs usually cause cancer cells to undergo rapid cell death through a direct cell membrane-disrupting effect, but some AMPs can trigger regulated cell death through intracellular signaling mechanisms. For example, previous research reports have indicated that some AMPs may promote K562 cancer cells death through intracellular calcium mechanisms, participation of free radicals and caspase-3 signaling pathway [16]. Previous research has also demonstrated that LfcinB disrupts cancer cell membranes, causing the loss of membrane integrity due to the formation of transmembrane pores that allow an uptake of the peptide into the cytoplasmic compartment of the cancer cell. The internalized LfcinB further colocalizes with the negatively charged mitochondria [25], [26] and results in cell death via an apoptotic process that involves the sequential generation of reactive oxygen species, the loss of Δφm, and the activation of the caspase cascade [15]. Furthermore, certain AMPs have been shown to be potent inhibitors of tumor angiogenesis, which is associated with tumor progression [27], [28].

Although temporin-1CEa treatment triggered a rapid cell death and resulted in striking morphological changes in MDA-MB-231 and MCF-7 cells, the results of the present study revealed an interesting concentration-related mechanism involved in temporin-1CEa-induced rapid cytotoxicity. The plasma membrane disruption during temporin-1CEa exposure is a progressive dose-response process with gradually increased permeability. After being exposed to peptides of lower concentrations, the plasma membrane became permeable to only small molecules, including PI (668 Da), EthD-1 (857 Da) or calcium. However, after being exposed to peptides of higher concentrations, the membrane was disrupted to be permeable for FITC-labeled temporin-1CEa (about 2310 Da) and LDH (140 kDa).

When cancer cells were treated with 20 µM FITC-labeled temporin-1CEa, the non-detectable intracellular green fluorescence suggested that temporin-1CEa at this concentration was prevented from influx into cancer cells. The membrane-bound peptides would induce membrane lipid asymmetry, membrane integrity disruption and enhancement of membrane permeability (as indicated by increased cell surface PS exposure and PI/EthD-1 uptake). Moreover, although temporin-1CEa of 20 µM was excluded from cancer cells, the peptides are still able to trigger intracellular events, including intracellular ROS and calcium ion elevation, transmembrane potential depolarization and loss of mitochondrial membrane potential. The calcium-related mechanisms have been identified to be involved in cell death induced by some certain antimicrobial peptide [29]. In our present study, the increased intracellular calcium concentration induced by 20 µM temporin-1CEa exposure was partlyly mediated by the endogenous calcium released from intracellular stores and have pivotal roles in temporin-1CEa-induced breast cancer cells death, although the detailed intracellular signaling pathway awaits further investigation.

When cancer cells were exposed to temporin-1CEa of higher concentrations, temporin-1CEa might induce membrane pore, or directly disrupt cell membranes to lysis. This membrane-disrupting effect resulted in PS exposure, membrane permeablization and even the release of cytoplasmic contents out of the cell, which ultimately leads to cell death. The membrane-bound temporin-1CEa might cause an influx of extracellular calcium into the intracellular compartment, which led to a rapid increase of intracellular Ca^2+^ and ROS concentration and a significant transmembrane potential depolarization.

The disrupted cell membrane induced by higher concentrations of temporin-1CEa may also permit extracellular peptides to be uptake into cells (as shown by increased intracellular green fluorescence from FITC-labeled temporin-1CEa) to initiate intracellular events and then cause cell death. Given the negative charge of mitochondrial membranes and their structural similarity with bacteria membrane, mitochondria are possibly the preferential intracellular structural target for internalized temporin-1CEa. Previous studies have indicated that AMPs disrupte mitochondrial potential and other mitochondrial functions [16], [30], [31]. In the present study, we hypothesized that the internalized temporin-1CEa together with the intracellular calcium overload triggered by endogenous calcium leakage from the intracellular calcium stores (such as endoplasmic reticulum) or calcium influxed from extracellular space, cause impairment of mitochondrial structure and function, including an opening of the mitochondrial permeability transition pore (PTP), thus triggered mitochondrial membrane permeabilization and the loss of ΔφM, and finally activation of cells death [32], [33]. However, temporin-1CEa at 20 µM was excluded from cancer cells. Whether the collapse of mitochondrial membrane potential induced by 20 µM temporin-1CEa is a result of increased intracellular Ca^2+^ production or a secondary result from an attack on metabolic pathways, await further investigation.

In addition, the research results in the present study also suggested that two human breast cancer cell lines, MCF-7 and MDA-MB-231, exerted different susceptibility and response manners to temporin-1CEa exposure. MCF-7 cells showed a higher susceptibility to the temporin-1CEa-induced cytotoxicity than MDA-MB-231 cells. Moreover, the membranes of MCF-7 cells seemed to be more vulnerable to temporin-1CEa exposure, as indicated by the higher permeability. The reasons for such sensitivity differences between these two cell lines might be due to the difference in membrane components, such as the phosphatidylserine, O-glycosylated mucins and cholesterol. These components are believed to promote electrostatic interactions with AMPs at the cancer cell surface [34]–[36] and thus in turn lead to various resistances to peptides-membrane interactions, including peptides binding on cell membrane and insertion through membranes into the intracellular spaces.

In summary, temporin-1CEa induced rapid cell death in two human breast cancer cell lines MDA-MB-231 and MCF-7, and MCF-7 cells was more vulnerable to peptides wxposure. This cytotoxic activity might be mediated by direct membrane-destruction and intracellular calcium–related mechanisms.
